# Static and Dynamic Assessments of a Sulfur-Triglyceride Composite for Antimicrobial Surface Applications

**DOI:** 10.3390/molecules30071614

**Published:** 2025-04-04

**Authors:** Shalini K. Wijeyatunga, Perla Y. Sauceda-Oloño, Nawoda L. Kapuge Dona, Bárbara G. S. Guinati, Katelyn M. Derr, Katelyn A. Tisdale, Ashlyn D. Smith, Andrew G. Tennyson, Rhett C. Smith

**Affiliations:** 1 Department of Chemistry, Clemson University, Clemson, SC 29634, USA; swijeya@g.clemson.edu (S.K.W.); psauced@g.clemson.edu (P.Y.S.-O.); dkapuge@g.clemson.edu (N.L.K.D.); bguinat@clemson.edu (B.G.S.G.); klinden@g.clemson.edu (K.M.D.); katisda@g.clemson.edu (K.A.T.); 2 Department of Materials Science and Engineering, Clemson University, Clemson, SC 29634, USA

**Keywords:** high sulfur content materials, inverse vulcanization, antimicrobial activity, gram-positive bacteria, gram-negative bacteria, fungal inhibition

## Abstract

Over 80 MT of elemental sulfur, a byproduct of fossil fuel desulfurization, are generated annually. This has spurred the development of high sulfur content materials (HSMs) via inverse vulcanization as a productive pathway towards sulfur utilization. In this study, we evaluate the antimicrobial performance of SunBG_90_, an HSM made from brown grease and sulfur, as tiles or infused into fabric squares. The static antimicrobial activity of SunBG_90_ tiles was assessed, revealing excellent efficacy against Gram-positive bacteria, with reductions of 96.84% for *Staphylococcus aureus* and 91.52% for *Listeria monocytogenes*. The tiles also exhibited strong antifungal activity, reducing *Candida auris* by 96.20% and mold (*fumigatus*) by 83.77%. In contrast, efficacy against Gram-negative bacteria was more variable, with moderate reductions for *Escherichia coli* (61.10%) and *Salmonella enteritidis* (62.15%), lower activity against *Campylobacter jejuni* and *Salmonella typhi*, and no effect on *Clostridium perfringens*. Under dynamic conditions, SunBG_90_-infused fabrics achieved a near-complete inhibition of *L. monocytogenes* (99.91%) and high reduction of *E. coli* (98.49%), along with a 96.24% inhibition of *Candida auris*. These results highlight the potential and limitations of SunBG_90_ for antimicrobial applications, emphasizing the need for further optimization to achieve consistent broad-spectrum activity.

## 1. Introduction

Over 80 MT of unutilized sulfur is produced annually by the desulfurization of fossil fuels, making sulfur utilization a critical goal for waste utilization. Preparing copolymers and composites with sulfur as the majority component, collectively termed high sulfur content materials (HSMs), has emerged as a potential way to utilize sulfur while creating a new class of materials with versatile applications [1,2,3,4,5,6,7,8,9,10,11,12,13,14,15,16,17,18,19,20,21,22,23,24,25,26,27,28,29,30,31,32,33,34,35,36,37,38]. HSMs are most commonly made by the inverse vulcanization of elemental sulfur with olefins, but more recent reports show that aryl halides, anisole derivatives, and even hydrocarbons or polymers featuring benzylic protons, are susceptible to reaction with elemental sulfur to produce HSMs. A wide range of organics have thus served as comonomers for the synthesis of HSMs, including plastic waste [39,40,41,42,43,44,45], plant oils [46,47,48,49,50,51], animal fats [40,41,52,53,54], wastewater sludge [55], terpenoids [4,42,56,57,58,59,60], polysaccharides [57,60,61,62,63,64], lignin [65,66,67,68,69,70], raw lignocellulosic biomass, and mixed-material waste. Prominent applications of HSMs include lithium–sulfur batteries [24,25,71,72,73,74,75,76,77], adhesives [46,47,78], and in separation/remediation agents [2,5,49,79,80,81,82,83,84,85]. The current report, however, will focus on the potential antimicrobial activity of HSMs.

Several recent reports indicate the potential of HSMs as antimicrobial agents. These materials are generally prepared by the inverse vulcanization of olefins with elemental sulfur. For example, coatings prepared using 1,3-diisopropenylbenzene (DIB) with embedded SiO_2_ nanoparticles exhibited an 81% inhibition of *E. coli* (a Gram-negative bacterium) and 75% inhibition of *S. aureus* (a Gram-positive bacterium) [86]. Another study on HSMs made from the inverse vulcanization of sulfur with dicyclopentadiene or DIB indicated up to a 99.9% reduction in *E. coli* and *S. aureus* viability [87]. A more systematic study on such materials revealed a dependence of efficacy on test temperature and the polymer’s glass transition temperature, thus offering insights into the potential to design materials for desired applications using structure–activity relationships [88,89]. Sulfur–nylon composites were similarly explored for activity against Gram-positive bacteria *S. aureus*, *B. subtilis*, and *S. epidermidis*, as well as Gram-negative bacteria *E. coli*, *P. aeruginosa*, and *K. pneumoniae*. Strong inhibition was only reported for some composites against *E. coli* [90].

The antimicrobial properties of HSMs made from the inverse vulcanization of more sustainable monomers have also been explored, including those to which other antimicrobial agents have been added. For example, Gentamicin-loaded HSMs from vegetable oils were shown to have increased efficacy against *P. aeruginosa* (a Gram-negative bacterium). In this study, the antibiotic was credited with killing the bacteria, while the sulfur-rich material was credited with helping to localize antibiotic accumulation at the infection site [91]. In another study, blends of poly(lactic acid) with an HSM made from sulfur and tung oil have demonstrated high efficacy against *S. aureus*, *B. subtilis*, *P. aeruginosa*, and *E.coli* [92].

Considerably less has been reported on the antimicrobial activity of HSMs against fungi than against bacteria. One study on an HSM made from sulfur and plant-derived urushiol indicated an inhibition efficiency greater than 80% against *S. aureus* and over 52% against the yeast *Saccharomyces cerevisiae* [93]. Another study combined tung oil with sorbic acid to form an HSM that was effective against *S. aureus, E. coli*, *B. subtilis*, *P. aeruginosa*, and *S. cerevisiae*. This HSM fully eliminated *S. aureus*, *B. subtilis*, *E. coli*, and *S. cerevisiae* in culture, while inhibiting over 90% of *P. aeruginosa* [94].

**Figure 1:**
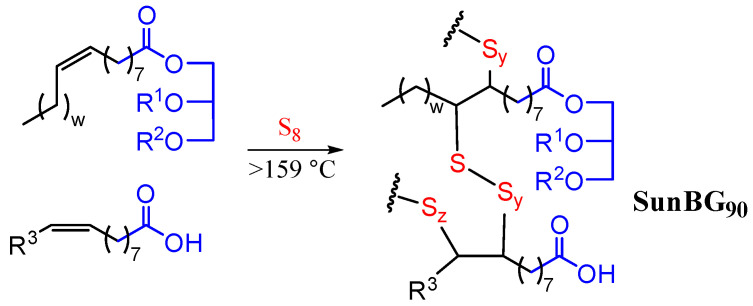
Structural representation of SunBG_90_. Sunflower oil is primarily composed of triglycerides and brown grease is a mixture of free fatty acids and monoglycerides, diglycerides, and triglycerides; R^1^ and R^2^ are H or fatty acid chains. R^3^ represents hydrocarbon chains to simplify the diagram.

The foregoing summary of studies involving the antimicrobial properties of HSMs highlights the potential for HSMs to exhibit broad efficacy against microorganisms, but also points to mixed results, sometimes even against the same test organisms. The composition and presence or absence of the supporting matrix, blend component, etc., all appear to influence the potential of HSMs against different types of organisms. In the current work, the antimicrobial efficacy of SunBG_90_ (Figure 1)—a well-studied animal fat-derived HSM made from sunflower oil (5 wt. %), brown grease (5 wt. %), and sulfur (90 wt. %) [54,95,96,97]—is evaluated. To provide a more complete picture of the antimicrobial ability of SunBG90 against microorganisms in different application settings, we studied inhibition under static and dynamic conditions. One part of this study used stationary tiles of SunBG90 to test against a range of pathogens representing Gram-negative bacteria, Gram-positive bacteria, and fungi (yeast and mold), according to the ASTM E 2180-18 protocol [98,99,100,101]. In the second part of this study, we infused SunBG90 into squares of fabric used in food preparation facility shoe coverings. We tested the ability of these fabric squares to inhibit select microorganisms of interest in food preparation or pharmaceutical facilities under dynamic conditions according to the ASTM E 2149-20 protocol [102,103,104,105].

## 2. Results and Discussion

### 2.1. Microbe Selection and Antimicrobial Properties of Static Hard Surfaces (ASTM E 2180-18)

#### 2.1.1. Preparation of Tile Test Samples and Test Organism Rationale

**Figure 2:**
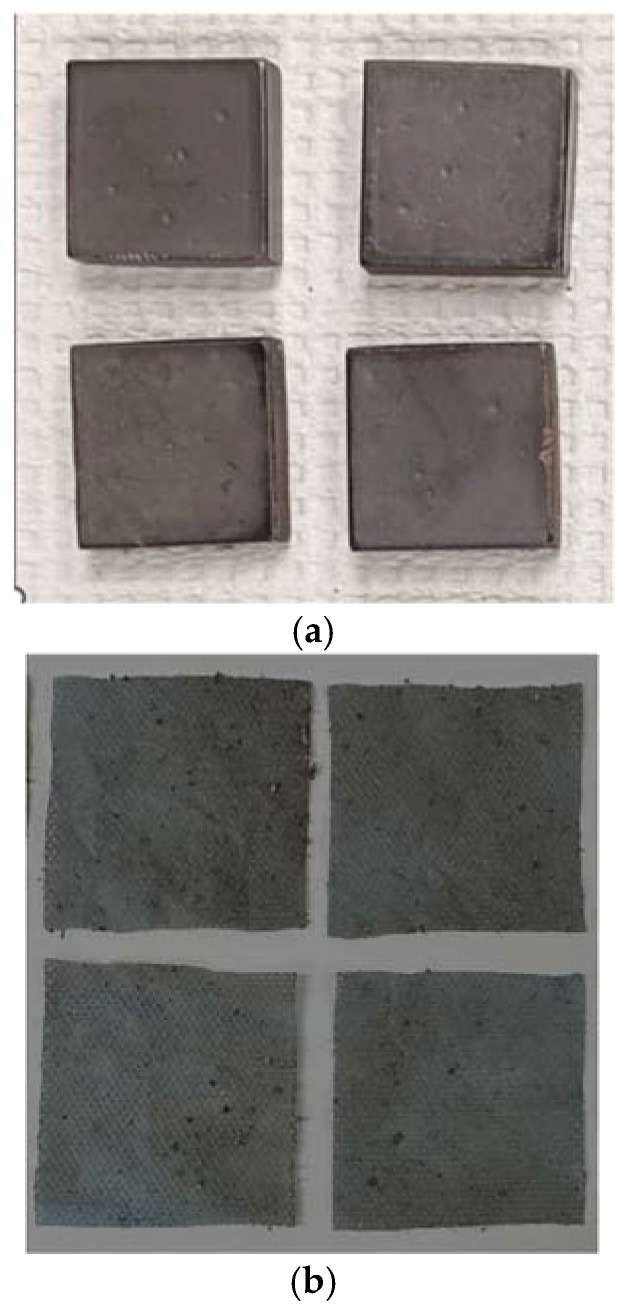
(**a**) Samples of SunBG_90_ tiles tested using the ASTM E 2180-18 method and (**b**) for antimicrobial activity assessment.

SunBG_90_ was prepared [54] and fabricated into tiles [97] according to the published procedures (Figure 2a).

**Figure 3:**
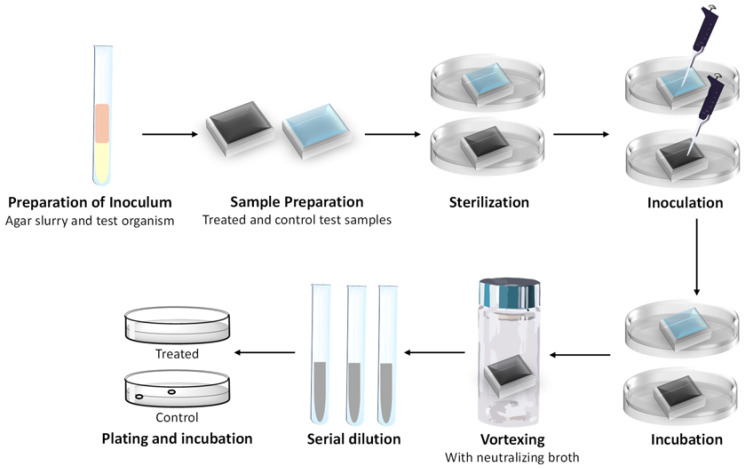
Steps of the ASTM E 2180-18 test protocol.

The antimicrobial performance of the SunBG_90_ tiles was evaluated using the ASTM E 2180-18 method (schematically represented in Figure 3), with results summarized in Table 1.

The assay assessed the reduction in viable counts of a range of microorganisms after 24 h of exposure to the material under static conditions. Microbes were selected from those studied in the aforementioned studies on the antimicrobial properties of HSMs or that pose particular threats to human health. For example, *Staphylococcus aureus* is a common Gram-positive bacterium responsible for skin infections, pneumonia, and bloodstream infections, including those caused by Methicillin-resistant *Staphylococcus aureus* (MRSA) [106,107,108]. Surfaces that inhibit *S. aureus* could prevent hospital-acquired infections. *Listeria monocytogenes* poses significant risks in food processing and storage environments, particularly in pregnant women, newborns, and immunocompromised individuals [109,110,111,112]. *Clostridium perfringens* is a spore-forming bacterium linked to food poisoning and gangrene [113,114,115,116]. *Salmonella enteritidis* [117,118,119,120] and *Campylobacter jejuni* (two strains are studied here) are major causes of gastroenteritis [121,122], and certain strains of *Escherichia coli* are pathogenic and cause severe gastrointestinal diseases [123,124,125,126]. These organisms are all critical targets in food industry settings. *Salmonella typhi* is the causative agent of typhoid fever, a significant public health concern in areas with inadequate sanitation [127,128,129,130]. Effective antimicrobial surfaces could help curb its transmission via contaminated water or surfaces in both healthcare and food service settings. In addition to bacteria, two fungi, *Candida auris* and *Aspergillus fumigatus*, were also evaluated in this study. *C. auris* is an emerging multidrug-resistant yeast known for causing invasive infections in healthcare settings [131,132,133,134]; so, effective antimicrobial surfaces can help prevent its rapid spread, particularly in hospitals and long-term care facilities. *A. fumigatus* is a mold responsible for invasive aspergillosis, especially in immunocompromised or COVID-19-afflicted individuals, which can lead to life-threatening conditions [135,136,137,138]. Surfaces that inhibit its growth are critical in healthcare and agricultural environments to reduce airborne spore exposure. 

#### 2.1.2. Efficacy Against Gram-Positive Bacteria

The tiles exhibited excellent antimicrobial activity against key Gram-positive bacteria. For example, *Staphylococcus aureus* (ATCC 6538) demonstrated a 96.84% reduction, and *Listeria monocytogenes* (ATCC 19115) showed a 91.52% reduction. This high efficacy is particularly significant given the clinical relevance of these pathogens frequently implicated in hospital-acquired infections.

#### 2.1.3. Variable Activity Against Gram-Negative Bacteria

In contrast to the consistently high activity against Gram-positive bacteria, the results against Gram-negative bacteria were more variable. *Salmonella enteritidis* (ATCC 13076) and *Escherichia coli* (ATCC 8739) showed similarly moderate reductions of 62.15% and 61.10%, respectively. However, for other Gram-negative organisms such as *Campylobacter jejuni* (ATCC 29428 and 33291) and *Salmonella typhi* (ATCC 14028), the reductions were lower—37.70%, 37.53%, and 32.87%, respectively. These differences may be attributed to the inherent structural complexity of Gram-negative cell envelopes, including the protective outer membrane that can impede the penetration or action of antimicrobial agents. The moderate efficacy observed suggests that while the SunBG_90_ disrupts these bacteria to some degree, the further optimization or addition of other agents to the tile formulation may be required to achieve higher levels of reduction across all Gram-negative species on these surfaces.

Interestingly, SunBG_90_ did not demonstrate any antimicrobial effect against *Clostridium perfringens* (ATCC 13124), with a reduction of 0%. *C. perfringens* is known for its resilient spore-forming capability and unique cell wall composition, which may confer intrinsic resistance to the antimicrobial mechanisms that are effective against other bacteria [109,113,114,115,116]. This finding indicates a limitation of the current formulation and underscores the need for additional investigations into the material’s spectrum of activity and possible modifications to target more resistant organisms.

#### 2.1.4. Efficacy Against Fungi

The antifungal activity of the tiles was evaluated using two representative fungal organisms. *Candida auris* (CDC B11903), an emerging yeast pathogen, experienced a 96.20% reduction, indicating that the material is highly effective against yeast forms. Similarly, the tiles reduced the viability of a mold, represented by *fumigatus* (ATCC 204305), by 83.77%. The robust performance against both yeast and mold suggests that the SunBG_90_ coating could possess broad-spectrum antifungal properties, which is especially valuable in environments where fungal contamination poses a risk.

### 2.2. Microbe Selection and Antimicrobial Properties of SunBG90-Infused Fabric Under Dynamic Conditions (ASTM E 2149-20)

#### 2.2.1. Preparation of SunBG_90_-Infused Fabric Squares

**Figure 4:**
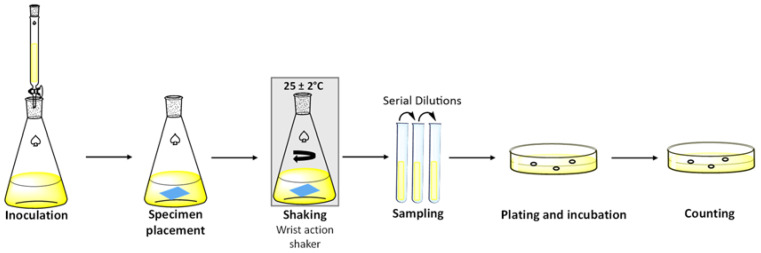
Steps of the ASTM E 2149-20 test protocol.

Fabric samples for testing in this study were prepared by cutting squares measuring 5.08 cm × 5.08 cm from commercial shoe coverings made of a spunbond–meltblown–spunbond (SMS) polypropylene material. Each fabric square was submerged separately in a 0.01 g/mL solution of SunBG_90_ in chloroform. The chloroform was then allowed to evaporate under ambient conditions (Figure 2b). This method leads to the infusion of 3 mg of material per square centimeter (19 mg per square inch) of fabric. The fabric squares maintained their flexibility and general appearance but with a slight darkening of the color after SunBG_90_ infusion. The antimicrobial performance of the SunBG_90_-infused fabric squares was assessed using the ASTM E 2149-20 method (steps are summarized in Figure 4), and the results are summarized in Table 2. In this method, a fabric square that has been infused with the agent is placed in growth media for the organism of interest and is in constant motion to test the ability of the fabric to reduce the organism under simulated movement as if the fabric was, for example, being worn as part of personal protective equipment for healthcare or industrial food service workers. Examples of the Gram-positive bacteria, Gram-negative bacteria, and a fungus against which SunBG_90_ tiles showed the greatest efficacy within each class of organism were selected for evaluation under these dynamic conditions.

#### 2.2.2. Activity Against Gram-Positive Bacteria

For *Listeria monocytogenes* (ATCC 19115), the infused fabrics achieved a substantial reduction of 99.91% after 8 h. This near-complete inhibition underscores the strong bactericidal effect of the antimicrobial agents incorporated into the fabric when challenged with a clinically significant Gram-positive organism. In contrast, *Staphylococcus aureus* (ATCC 6538) was not reduced under the same conditions, suggesting that differences in bacterial cell wall composition or resistance mechanisms may limit the efficacy of the antimicrobial compounds in this context.

#### 2.2.3. Activity Against Gram-Negative Bacteria

Among Gram-negative bacteria, the results were similarly selective. *Escherichia coli* (ATCC 8739) was highly susceptible to the treatment, exhibiting a 98.49% reduction after 8 h. On the other hand, *Salmonella enteritidis* (ATCC 13076) displayed no reduction, which may be due to variations in the outer membrane composition or other intrinsic resistance factors that hinder the activity of the antimicrobial agents released from the fabric.

#### 2.2.4. Activity Against Yeast

The infused fabrics also demonstrated strong antifungal properties. *Candida auris* (CDC B11903) showed a 96.24% reduction after 8 h, indicating that the fabric formulation is highly effective against this problematic yeast. This robust antifungal activity is significant given the challenges of controlling *C. auris* in healthcare and other high-risk environments.

### 2.3. Summary of Antimicrobial Properties

The results obtained from the SunBG_90_-coated tiles and the SunBG_90_-infused fabrics provide valuable insights into the antimicrobial performance of this novel material across different substrates and testing conditions.

In the tile study (ASTM E 2180-18), the SunBG_90_ coating demonstrated high efficacy against key Gram-positive bacteria. Both *Staphylococcus aureus* and *Listeria monocytogenes* exhibited reductions exceeding 90%, and the material also showed potent antifungal activity against the pathogenic yeast *Candida auris* and pathogenic mold *Aspergillus fumigatus*. However, the activity against Gram-negative bacteria was more variable, with moderate reductions observed for *Escherichia coli* and *Salmonella enteritidis*, and considerably lower efficacy against *Campylobacter jejuni* and *Salmonella typhi*. Notably, no antimicrobial effect was observed against *Clostridium perfringens*. These outcomes suggest that while the SunBG_90_ coating is highly effective against many clinically relevant organisms, its activity may be hindered by structural features such as the outer membranes characteristic of Gram-negative bacteria or the inherent resilience of spore-formers like *C. perfringens*.

The antimicrobial performance exhibited a pronounced time-dependent effect in the fabric study (ASTM E 2149-20), which employs dynamic agitation in a liquid medium. After 8 h of exposure, the fabrics achieved the near-complete inhibition of *Listeria monocytogenes* (99.91%) and a high reduction in *Escherichia coli* (98.49%). The antifungal performance was similarly robust against *Candida auris* (96.24%). In contrast, the fabric failed to reduce Staphylococcus aureus and *Salmonella enteritidis* under the same conditions. This selective efficacy could be attributed to differences in the release kinetics of the active compounds from the fabric matrix or to variations in microbial cell wall composition that influence the interaction with the antimicrobial agents.

When comparing the two substrates, several factors emerge. The tiles, with their static application of the antimicrobial coating, likely offer a more uniform and immediately available active surface, which explains the consistent performance against many Gram-positive organisms and fungi. Conversely, the efficacy of the fabric relies on the diffusion and release of microbes over time—a process that seems highly effective for certain organisms like *L. monocytogenes* and *E. coli* but less so for others such as *S. aureus* and *S. enteritidis*. This discrepancy may be due to differences in the physical and chemical environments provided by a rigid surface versus a flexible, porous matrix.

## 3. Materials and Methods

### 3.1. Testing of Tiles According to ASTM E 2180-18

#### 3.1.1. Sample Preparation

The antimicrobial activity of polymeric tile surfaces was evaluated using ASTM E2180-18, a standard method for determining the activity of incorporated antimicrobial agents in hydrophobic or polymeric materials. The test material, labeled SunBG_90_ (as previously reported [54], elemental analysis calculated C 3.85, H 1.00, S 90.00 and found C 5.17, H 0.40, S 92.33), was tested against ten microorganisms. Control tiles of identical composition without antimicrobial treatment were used as negative controls.

Each tile sample was prepared as a uniform 2.5 × 2.5 cm section according to the reported procedure [97] and placed into sterile Petri dishes in triplicate for each tested microorganism.

#### 3.1.2. Microorganisms and Growth Conditions

The challenge microorganisms used in this study were sourced from ATCC and included the following:

*Campylobacter jejuni* (ATCC 33291, ATCC 29428);
*Aspergillus fumigatus* (ATCC 204305);
*Salmonella typhi* (ATCC 14028);
*Salmonella enteritidis* (ATCC 13076);
*Clostridium perfringens* (ATCC 13124);
*Listeria monocytogenes* (ATCC 19115);
*Candida auris* (ATCC CDC B11903);
*Staphylococcus aureus* (ATCC 6538);
*Escherichia coli* (ATCC 8739).

Bacterial cultures were grown for 18–72 h and fungal cultures were grown for 5–7 days on appropriate media at required incubation conditions:

Aerobic incubation (30–35 °C) for most bacteria;
Anaerobic incubation (30–35 °C) for *C. perfringens*;
Microaerophilic incubation (37 ± 2 °C) for *C. jejuni*;
Fungal incubation (20–25 °C) for *A. fumigatus* and *C. Auris*.

Cultures were adjusted to 1–5 × 10^8^ CFU/mL using a spectrophotometer before being incorporated into the testing medium.

#### 3.1.3. Preparation of Inoculated Agar Slurry

A thin-layer agar slurry method was used to ensure uniform microbial contact with the tile surface. The slurry was prepared by dissolving 0.85 g NaCl and 0.3 g agar-agar in 100 mL of deionized water. For each organism, a separate agar slurry was prepared and equilibrated at 45 °C. The standardized microbial suspension was added to the molten agar to achieve a final concentration of 1–5 × 10^6^ CFU/mL.

#### 3.1.4. Experimental Procedure

##### Surface Preparation

The tile surfaces were pre-wet using sterile 0.85% saline applied with a cotton swab to promote the even distribution of the inoculum.

##### Inoculation and Incubation

A 0.5–1 mL aliquot of the inoculated agar slurry was pipetted onto the test and control tiles.

The slurry was allowed to gel, ensuring consistent contact with the tile surface.

The samples were incubated at their respective contact temperatures for 24 h.

##### Microbial Recovery and Enumeration

After 0 h (immediately after inoculation) and 24 h (post-incubation), microorganisms were recovered by transferring the tile surfaces into 120 mL sterile neutralizing broth to achieve an initial 1:10 dilution.

The tiles were vortexed for 1 min to release attached microorganisms.

Serial dilutions were performed, and colony-forming units (CFU/mL) were determined by plating on appropriate media.

#### 3.1.5. Controls and Quality Assurance:

Purity Control: Each microorganism was streaked and incubated to confirm identity via Gram staining and colony morphology.

Sterility Control: All reagents, diluents, and growth media were tested for sterility prior to use.

Growth Promotion Test: A low-inoculum control (<100 CFU) was plated to confirm media viability.

#### 3.1.6. Data Analysis

Microbial reduction was calculated as the percent reduction and log reduction relative to the untreated controls using the following equations:

$$Percent\;Reduction=\left(\frac{a-b}{a}\right)\times 100\;{Log}_{10}\;Reduction={Log}_{10}a-{Log}_{10}b$$

where the variables are defined as follows:

*a* = Antilog of the geometric mean CFU/mL from the control samples.

*b* = Geometric mean CFU/mL from the treated samples.

#### 3.1.7. Interpretation of Results

Tiles with >90% bacterial reduction were considered highly effective, while those with <50% reduction were considered minimally effective.

### 3.2. Testing of Fabric Squares According to ASTM E 2149-20

#### 3.2.1. Sample Preparation

SunBG_90_ was prepared according to the reported procedure by heating sulfur, brown grease, and sunflower oil [97]. Squares measuring 5.08 cm × 5.08 cm were cut from commercial shoe covers (KIMTECH Shoe Covers, Model 69254). Each fabric square was submerged separately in a solution containing approximately 0.01 g/mL of SunBG_90_ dissolved in chloroform. After the chloroform had completely evaporated, the squares were removed and weighed before and after infusion.

Textile fabric squares were tested for antimicrobial activity according to ASTM E2149-20, which evaluates antimicrobial activity under dynamic contact conditions. Five fabric samples, each treated with antimicrobial agents according to the above procedure, were obtained. Untreated control fabric samples with an identical composition were also included. Each sample was weighed to 1.0 ± 0.1 g before testing.

#### 3.2.2. Bacterial Culture Preparation

Challenge microorganisms were sourced from standard culture collections and included the following:

*Listeria monocytogenes* (ATCC# 19115);

*Escherichia coli* (ATCC# 8739);

*Staphylococcus aureus* (ATCC# 6538);

*Salmonella enteritidis* (ATCC# 13076);

*Candida auris* (CDC# B11903).

Bacterial cultures were grown in Tryptic Soy Broth (TSB) at 35 ± 2 °C for 18 h prior to testing. The cultures were then diluted in Phosphate Buffer Solution (PBS) with a 0.01% wetting agent to achieve a final working concentration of 1.5–3.0 × 10^5^ CFU/mL, as determined by absorbance measurements at 475 nm on a spectrophotometer.

#### 3.2.3. Experimental Procedure

Each fabric sample was placed in a sterile 250 mL screw-cap Erlenmeyer flask containing 50 ± 0.5 mL of the bacterial suspension. The flasks were secured onto a wrist-action shaker and agitated at the maximum stroke for 8 h at 25 ± 2 °C to ensure dynamic contact between the microorganisms and the fabric surface.

After the exposure period, aliquots were removed from each flask, serially diluted, and plated on selective agar media, including the following: 

Tryptic Soy Agar (TSA) (general enumeration);

MacConkey Agar (Gram-negative bacteria);

Mannitol Salt Agar (*S. aureus*);

Xylose Lysine Deoxycholate (XLD) Agar (*Salmonella*);

Sabouraud Dextrose Agar (*C. auris*).

Plates were incubated aerobically for 48 h at 35 ± 2 °C for *L. monocytogenes*, *E. coli*, *S. aureus*, and *S. enteritidis*, and 20–25 °C for *C. auris*. Colony-forming units (CFU) were counted, and the percent reduction in bacterial population was calculated relative to the untreated control using the following formula:

$$Percent\;Reduction=\left(\frac{B-A}{B}\right)\times 100\;{Log}_{10}\;Reduction={Log}_{10}B-{Log}_{10}A$$

where the variables are defined as follows:

*A* = CFU/mL for the treated fabric after contact time;

*B* = CFU/mL for the untreated control.

#### 3.2.4. Control and Quality Assurance

Sterility Control: All reagents, buffers, and neutralizers were tested to confirm sterility before use.

Purity Control: Each bacterial culture was verified via Gram staining and colony morphology prior to testing.

Growth Promotion Test: Less than 100 CFU of each challenge microorganism was inoculated into its respective growth media to confirm viability.

## 4. Conclusions

Our investigation on the antimicrobial properties of SunBG_90_ reveals that the material’s efficacy is strongly influenced by both its substrate form and the testing conditions. Under static conditions (ASTM E 2180-18), SunBG_90_-coated tiles demonstrated outstanding activity against key Gram-positive pathogens, achieving reductions of 96.84% for *Staphylococcus aureus* and 91.52% for *Listeria monocytogenes*. The tiles also exhibited robust antifungal activity, with a 96.20% reduction for *Candida auris* and an 83.77% reduction for *A. fumigatus*. In contrast, antimicrobial performance against Gram-negative bacteria was more variable, with moderate reductions observed for *Escherichia coli* (61.10%) and *Salmonella enteritidis* (62.15%), and lower efficacy against *Campylobacter jejuni* (approximately 37.7%) and *Salmonella typhi* (approximately 32.9%), while no effect was detected against *Clostridium perfringens*. Under dynamic conditions (ASTM E 2149-20), SunBG_90_-infused fabric squares achieved the near-complete inhibition of *L. monocytogenes* (99.91%) and a high reduction in *E. coli* (98.49%), along with strong antifungal efficacy against *Candida auris* (96.24%), although no reduction was observed for *S. aureus* and *Salmonella enteritidis*. These findings underscore that the antimicrobial performance of SunBG_90_ is substrate-dependent, with static tiles providing a uniformly active surface and dynamic fabrics relying on the controlled release of active agents. It should be noted that the potential of SunBG_90_ to irritate skin is as-yet unknown. Current fabrics are intended for use as shoe coverings in food preparation or pharmaceutical facilities, where these fabrics are not exposed to skin directly, but additional studies would be needed for any skin-contact applications. This study highlights both the promise and the limitations of SunBG_90_, emphasizing the need for further formulation optimization to achieve consistent, broad-spectrum antimicrobial efficacy.

## Figures and Tables

**Table 1:** Antimicrobial efficacy of SunBG_90_ tiles tested by ASTM E 2180-18 method.

Organism	ATCC # or CDC#	Classification	Time (h)	Reduction (%)
*L. monocytogenes*	19115	Gram-positive Bacteria	24 h	91.52
*S. aureus*	6538	Gram-positive Bacteria	24 h	96.84
*C. perfringens*	13124	Gram-positive Bacteria	24 h	0
*S. enteritidis*	13076	Gram-negative Bacteria	24 h	62.15
*E. coli*	8739	Gram-negative Bacteria	24 h	61.10
*C. jejuni*	29428	Gram-negative Bacteria	24 h	37.70
*C. jejuni*	33291	Gram-negative Bacteria	24 h	37.53
*S. typhi*	14028	Gram-negative Bacteria	24 h	32.87
*C. auris*	CDC B11903	Yeast	24 h	96.20
*A. fumigatus*	204305	Mold	24 h	83.77

**Table 2:** Antimicrobial efficacy of SunBG_90_-infused fabrics tested by ASTM E 2149-20 method.

Organism	ATCC # or CDC #	Classification	Time (h)	Reduction (%)
*L. monocytogenes*	19115	Gram-positive Bacteria	1 h	13.35
*L. monocytogenes*	19115	Gram-positive Bacteria	8 h	99.91
*S. aureus*	6538	Gram-positive Bacteria	8 h	0
*E. coli*	8739	Gram-negative Bacteria	8 h	98.49
*S. enteritidis*	13076	Gram-negative Bacteria	8 h	0
*C. auris*	CDC B11903	Yeast	8 h	96.24

## Data Availability

Data are provided in the article or can be requested from the corresponding authors.

## Abbreviations

The following abbreviations are used in this manuscript:

HSM	High Sulfur Content Material
SunBG_90_	Composite made from 90 wt. % sulfur, 5 wt. % brown grease, and 5 wt. % sunflower oil
DIB	1,3-Diisopropenylbenzene
SMS	Spunbond–Meltblown–Spunbond
ASTM	American Society for Testing and Materials
